# Enhancing Apoptosis in Glioblastoma Through Hybrid Nanoparticle-Mediated Resveratrol Delivery: *In Vitro* Cytotoxicity and *in Vivo* Pharmacokinetic Study

**DOI:** 10.34172/apb.46117

**Published:** 2026-05-09

**Authors:** Swati Devendra Raysing, Ashish Prakash Gorle

**Affiliations:** ^1^Department of Quality Assurance, R.C. Patel Institute of Pharmaceutical Education & Research, Near Karwand Naka, Shirpur 425405, Dist. Dhule, M.S; India; ^2^Department of Pharmaceutics, R.C. Patel Institute of Pharmaceutical Education & Research, Near Karwand Naka, Shirpur 425405, Dist. Dhule, M.S; India

**Keywords:** Resveratrol, Cytotoxicity, Cellular apoptosis, ROS estimation, Cell cycle kinetics, MMP estimation, Lipopolymeric hybrid nanoparticles, Brain delivery

## Abstract

**Purpose:**

Glioblastoma multiforme (GBM) is the most prevalent malignant primary brain tumor of the central nervous system (CNS), with limited therapeutic success due to challenges such as the blood-brain barrier and poor drug bioavailability. Resveratrol (RSV), a natural polyphenol with known anticancer activity, shows promise against GBM but suffers from low systemic absorption and limited brain targeting. This study investigates the therapeutic potential of RSV-loaded lipopolymeric hybrid nanoparticles (RSV-LPHNs) as a novel delivery platform to enhance RSV’s efficacy in GBM treatment.

**Methods:**

This study evaluated the anticancer and targeting efficiency of RSV-LPHNs through a series of in vitro and in vivo assays. The potential of RSV-LPHNs against GBM was investigated by assessing Cellular Apoptosis, ROS Estimation, cell cycle kinetics, MMP Estimation with Flow Cytometry- U87 and DNA Fragmentation Assay. Furthermore, in vivo pharmacokinetic studies in rat were performed followed by brain distribution study.

**Results:**

In U87 glioblastoma cells, RSV-LPHNs induced apoptosis via increased reactive oxygen species (ROS) production and disrupted mitochondrial membrane potential (MMP), confirmed by flow cytometry. In vivo pharmacokinetic studies in rats showed that RSV-LPHNs achieved a significantly higher AUC (0–∞) (94.58 µg•h/mL) compared to plain RSV (10.68 µg•h/mL), resulting in a 3.77-fold improvement in relative bioavailability. Brain distribution studies demonstrated a notable increase in brain AUC, from 7.11 to 47.20 µg•h/mL, indicating a 2.28-fold enhancement in brain.

**Conclusion:**

By improving RSV’s cellular uptake, apoptotic effect, and brain delivery, RSV-LPHNs may represent a viable and effective neurotherapeutic platform for combating glioblastoma multiforme.

## Introduction

 Glioblastoma multiforme (GBM) is the most prevalent malignant primary brain tumor of the central nervous system (CNS), making almost 48.06 percent of malignant tumors along with 14.05 percent of other CNS tumors.^1^ This is also one of the most aggressive cancers. GBM patients only have a 15-month median overall survival (OS).^2^ GBM presents major treatment hurdles due of its characteristics, which include diffuse infiltration, considerable cellular heterogeneity, fast proliferation, necrosis, and neovascularization. GBM is a high grade glioma (grade IV-malignant) that proliferates via astrocytic type of glial cells.^3^ Here a rate of occurrence of GBM varies from 3.18 instances per one lacs person per years^4,5^ to 4.18 reports per one lacs person per years,^6^ rendering it challenging to characterize precisely. According to the 2016 WHO classification, glioblastoma is a grade IV tumor categorized into four types based on genetic and histological features. The most common is isocitrate dehydrogenase (IDH)-wildtype, typically seen in older adults and associated with poor prognosis. IDH-mutant GBM arises from lower-grade gliomas in younger patients and generally has a better outcome.^6^

 The barrier between blood and brain (BBB) is a hurdle while targeting therapeutic active agent towards brain. Composed of tightly joined endothelial cells, enzymes, receptors, and transporters for efflux including P-Glycoprotein (P-gp), it functions both as a physical and biochemical barrier. The BBB restricts the entry of nearly 98% of small molecules and almost all drugs exceeding 500 Da in molecular weight. As a result, many anticancer drugs fail to penetrate it effectively. P-gp, an ATP-dependent efflux pump, actively removes xenobiotics and therapeutic compounds, limiting drug accumulation in the brain. Most anticancer drugs are either too large, hydrophobic, or recognized as P-gp substrates, reducing their therapeutic efficacy. This active efflux mechanism also exists in tumor vasculature, compounding the challenge. Overcoming this barrier is critical for effective brain cancer treatment. Hence, advanced strategies like nanoparticle-based delivery systems are being explored to bypass or modulate the BBB. These difficulties have made the development of innovative therapeutic strategies that can get around the drawbacks of traditional therapies and enhance medication delivery to tumor locations imperative.^7,8^

 Nanodrug delivery is an emerging approach for targeted treatment of brain tumors. It offers a solution to the limitations of traditional therapies like chemotherapy and invasive procedures. By overcoming biological barriers such as the blood–brain barrier, nanotechnology enhances drug delivery to tumor sites. Nanodevices can penetrate brain tissue, avoid immune detection, and deliver drugs specifically to cancer cells. These advancements improve treatment precision and reduce damage to healthy tissue. The ability to bypass systemic resistance makes nanotherapy a promising cancer treatment strategy. Overall, it represents a significant leap toward more effective brain tumor management.^8,9^ Nanoparticles, owing to their suitable size, modifiable surfaces, encapsulation capacity for water soluble and insoluble agents, can be engineered to avoid biological barriers and achieve site-specific targeting of drug. Solid-lipid nanoparticles (SLNs), nanostructured carriers for lipids (NLCs), and lipo-polymeric hybrid nanocarriers (LPHNs) are novel platforms which employ both passive-active delivery mechanisms to transport therapeutics directly towards specific tissue. Although SLNs and NLCs were extensively applied in glioblastoma (GBM) therapies—facilitating the delivery of macromolecules, genetic material, oligo-nucleotides, si-RNA, enzymes—they still face fewer drawbacks. These are extremely crystalline lipid matrix frequently results in reduced loading of drug with drug ejection at the time of storage. Even though they can alleviate some of the shortcomings of SLNs, their physical characteristics may become unstable over time due to intricate manufacturing methods.

 Resveratrol, a stilbene-derived active agent that exhibits trans and cis forms. Structurally, it’s made up of two phenolic rings connected by a double bond of styrene, forming 3,4′,5-trihydroxystilbene.^10,11^ These goods demonstrate the increasing market demand for nutraceuticals based on resveratrol, highlighting the value of current research in overcoming contemporary trends in medicinal sector. Furthermore, resveratrol has been demonstrated to higher sensitivity of tumors to chemotherapeutic agents, hence overcoming treatment resistance in these cells.^12^ Numerous human tumor cells have been shown to be cytotoxically affected by resveratrol in vitro.^10^

 By combining the advantages of lipid- and polymer-based systems, LPHNs, on the other hand, get beyond these restrictions and provide better stability, increased drug-loading efficiency, and regulated drug release.^13,14^ Additionally, LPHNs are more functionally versatile and have improved biocompatibility, which makes them especially well-suited to successfully distribute therapeutic drugs in GBM therapies and pass the blood-brain barrier.^15,16^ These characteristics make LPHNs a more reliable and effective platform for sophisticated drug delivery applications than SLNs and NLCs. LPHN is an excellent choice for targeting of the drug in the GBM because of all of these characteristics.^17,18^

 Our research group has already developed and evaluated *in-vitro* cytotoxicity study for LPHN in GBM tissues and very outstanding results were observed.^19,20^ The present investigation aims to elucidate the cytotoxicity, cellular uptake, apoptosis induction, ROS generation, mitochondrial dysfunction, DNA fragmentation, and pharmacokinetic behaviour.

## Materials and Methods

###  Materials

 The solvent Methanol was purchased from Merck Specialties Pvt. Ltd, Mumbai, India. Ortho-phosphoric was ordered from Chemtex Speciality Limited, Mumbai, India. Trypsin, EDTA, ethanol was received after order from Hi-Media Pvt. Ltd., Mumbai. Propidium Iodide (PI) JC1 staining buffer was purchased from Elabscience, Houston, Texas, USA. Fetal Bovine Serum (FBS) and DMEM medium (Dulbecco’s Modified Eagle Medium-AT149-1L) were purchased from Himedia India. Male and female species of Sprague Dawley Rats were obtained from Lacsmi Biofarms, Alephata, Tal. Narayangaon, Dist. Pune, India. U87 Cell lines were Procured from NCCS Pune.

###  Methods

####  Cellular Apoptosis

 U87 cells lines were treated with IC_50_ dose of the sample then washed twice with cold phosphate buffer solution to re-suspend in1 time binding buffer at a concentration of 1 × 10^6^ cells/ml and cells were divided into the groups of Unstained cells, Control group, only Annexin, Propidium iodide only and Treatment sample. Annexin V FITC and PI were added to respective labelled tubes. After vortexing the sample with incubation up to fifteen minutes at RT, buffer (as a binder) was put in each sample tube.^21^

####  Reactive Oxygen Species Estimation

 U87 cells were seeded into 6-well plates at a density of 5,000 to 8,000 cells per well in 1 mL of DMEM (10% FBS and 1% antibiotic solution). The cells were allow to incubate up to 24 hours at RT in a humidified surrounding with 5 percent of CO_2_. After incubating, the spent media was then discarded to replace with another freshly prepared prior to treatment. Cells were then exposed to different dilutions of concentrations and incubated for an additional 24 hrs. Then the medium was removed, and cells were trypsinized with help of trypsin-EDTA, collected into 1.5 mL microcentrifuge tubes, and washed once with 500 μL of chilled PBS. The resulting cellular pellets were re-suspended to 100 μL of PBS containing 2 μM DCFDA and tested on flow cytometer (BD FACS Calibur, USA) within one hour. Data acquisition and analysis were done using Flowing Software version 2.5.1.^22^

####  Elucidating Cell Cycle Kinetics Through Flow Cytometry

 Cellular culture was seeded and then treated with different concentrations of the test samples (as detailed in the Excel sheet) upto 24 hours. Following incubation, the cells were detached using trypsin and transferred into 1.5 mL microcentrifuge tubes. They were washed once with 500 µL of cold phosphate-buffered saline (PBS). Roughly 1 × 10⁶ cells were then suspended in 100 µL of PBS and gently vortexed to ensure a uniform single-cell suspension with minimal aggregation. To fix the cells, the suspension was slowly added dropwise into 900 µL of pre-chilled 70% ethanol in centrifuge tubes and incubated at 4°C for a minimum of 2 hours. Fixed samples could be kept in 70% ethanol at 4°C for several weeks if necessary. Post-fixation, cells were centrifuged, and the resulting pellet was resuspended in 500 µL of a Propidium Iodide (PI) staining solution composed of PBS with 0.1% Triton X-100, 10 µg/mL PI, and 100 µg/mL RNase A (DNase-free). The samples were incubated in the dark for 30 minutes at room temperature or for 10 minutes at 37°C. Flow cytometric analysis was then conducted using a BD FACS Canto II system.^23^

####  MMP Estimation 

 The cultured U87 were made in contact with to the IC_50_ concentrations of the standard as well as test formulations, after 24-hour incubation period. After incubation, culture media was discarded, and cells were harvested using trypsin-EDTA (HiMedia TC038M). The collected cells were transferred into 1.5 mL microcentrifuge tubes and washed once with 500 μL of chilled PBS. Finally, cells pellet was dispensed in 400 µl JC1 staining buffer with 2μM JC-1 dye (Elabsciences-Cat no.- A301A) and the samples were acquired in Flow Cytometer (BD FACS Calibur, USA) within 1 hour.^23^

####  DNA Fragmentation Assay

 U87 cells (10,000 cells per well) were seeded into a 96-well plate and allowed to adhere for 24 hours in Dulbecco’s Modified Eagle Medium (DMEM, AT149-1L) containing 10% Fetal Bovine Serum (FBS, HiMedia RM10432) and 1% antibiotic solution. The incubation was carried out at 37°C in a humidified incubator with 5% CO_2_. After 24 hours, the cells were exposed to the IC₅₀ concentrations of various formulations and incubated for an additional 24 hours. Following treatment, dead cells were harvested, and genomic DNA was isolated using a standard protocol. The extracted DNA was subjected to electrophoresis on a 1% agarose gel at 90 V for 30 minutes. The resulting gel was imaged using a documentation system (CAMAG Reprostar 3 with Canon 1300D).^24^

####  In-Vivo Study in Rat (Pharmacokinetic Study)

 Pharmacokinetic studies were performed in male and female Sprague Dawley rats (8–12 weeks old, 220–250 g). Animals were fasted for 12 h prior to the study and divided into two groups (n = 6 per group): (i) Resveratrol API administered as a suspension and (ii) Resveratrol novel formulation administered as a lipid–polymer hybrid nanoparticle (LPHN) dispersion. Both groups received a single oral dose of 20 mg/kg of Resveratrol. Blood samples were collected at 0.5, 1, 2, 4, 6, 8, 12, and 24 h post-dosing via the tail vein. Plasma was separated immediately by centrifugation, and proteins were precipitated with acetonitrile (1:2 v/v). The supernatant was subjected to HPLC analysis (Agilent 1100 with auto-sampler and Chemstation software) using a C18 column (4.6 × 250 mm, 5 μm). The mobile phase consisted of methanol and 0.1% ortho-phosphoric acid (80:20 v/v) at a flow rate of 0.7 mL/min, with the column maintained at 28 °C and detection at 318 nm.^25^

####  Brain Distribution Study

 For biodistribution study, another animals were dosed at 20mg/kg p.o. (single dose), animals were euthanized at 6 hours of time interval. Brain tissue was excised, washed with saline water and finely minced into phosphate buffer solution (PBS) and homogenate was centrifuged at 3000g; supernatant was collected and further processed for HPLC analysis for estimation of Resveratrol concentration.^26^

####  Statistical Analysis

 All experiments were performed in triplicate (n = 3) unless otherwise stated. Data are expressed as mean ± standard deviation (SD). Statistical analyses were carried out using GraphPad Prism software Comparisons between two groups were performed using unpaired Student’s t-test, while comparisons among more than two groups (e.g., ROS, DNA fragmentation assays) were analyzed using one-way ANOVA followed by Tukey’s post hoc test. For pharmacokinetic studies, parameters between RSV suspension and RSV-LPHNC groups (n = 6 per group) were compared using unpaired Student’s t-test. A *P*-value < 0.05 was considered statistically significant. Significance levels are indicated as *P* < 0.05, *P* < 0.01.

## Results and Discussion

###  Cellular Apoptosis with Flow Cytometry

 Cellular apoptosis in U87 glioblastoma cells which were treated with test sample (RSV-LPHNC) at a dose of IC_50_ was analyzed using flow cytometry. The results are presented in Figure 1 in which U87 glioblastoma cells were categorized in 5 categories: unstained, control, Annexin V only, PI only, and treated. It’s then observed that unstained group cells showed nonspecific fluorescence due to minimal background signal. The PI only and Annexin only treated cell lines showed selective binding to necrotic and early apoptotic cells, respectively. The minimal apoptosis with healthy cells were found in uncontrolled group in lower left quadrant. On other hand, RSV-LPHNC-treated cells showed significant shift of dot distribution towards upper right and left quadrants demonstrating enhancement in late apoptotic (Annexin V+/PI+) and early apoptotic (Annexin V+/PI–) cells, respectively. This observation lays down the foundation of RSV-LPHNC induces apoptosis in U87 cells, validating its potential cytotoxic and anti-cancer activity. The staining with accompanied with Annexin V-FITC along with PI effectively differentiated live, early apoptotic, late apoptotic, and necrotic cell populations, supporting the reliability of this method for apoptosis detection.

**Figure 1:**
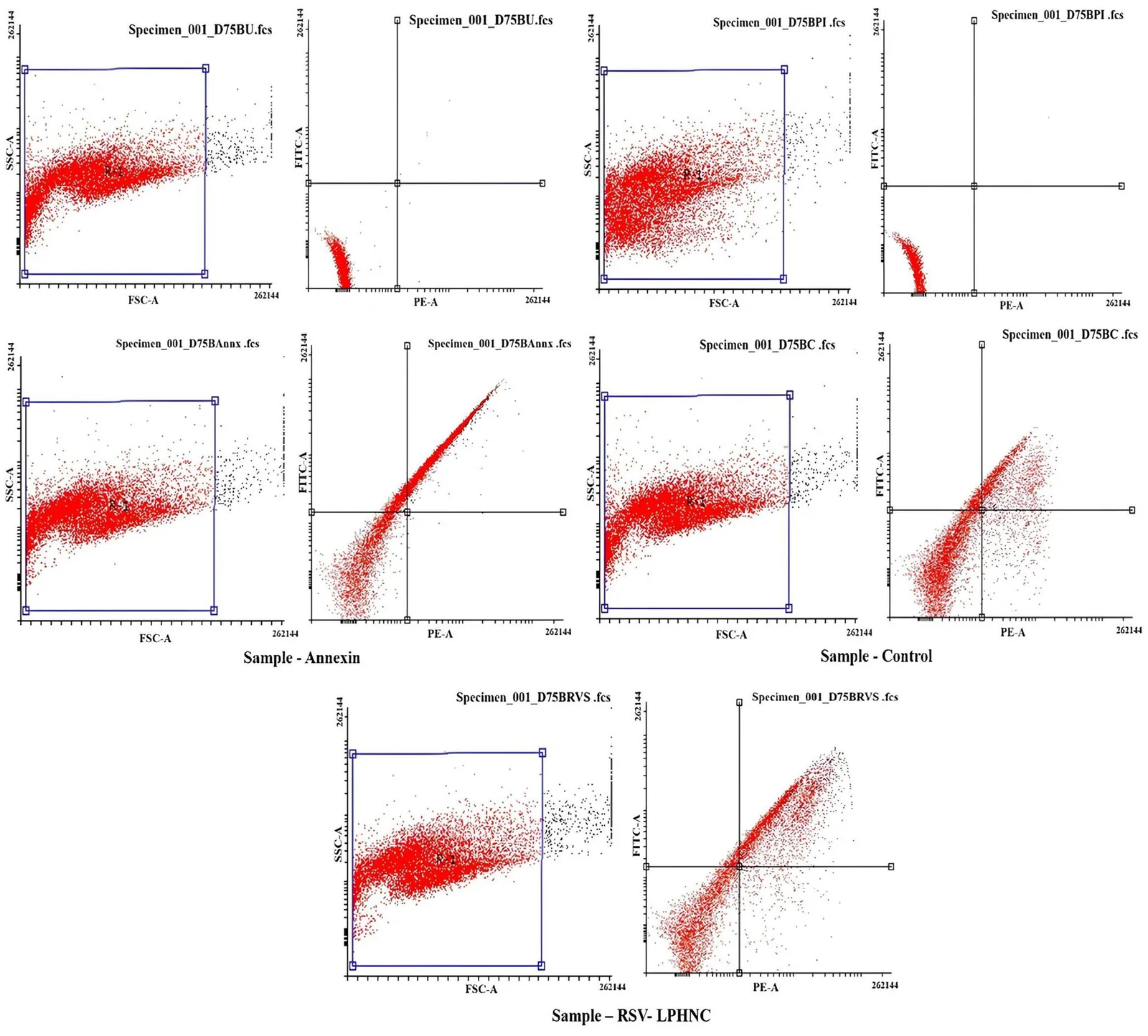
Cellular apoptosis results with flow cytometry

Figure S1 (A) presents the results of a cellular apoptosis assay conducted on U87 cells, comparing untreated control cells with cells treated using RSV-LPHNC. Based on the analysis results, it was observed that after 15 min of incubation post-treatment, control exhibited 56% viable cells, 24% in the early apoptotic stage, 16.1% in the late apoptotic stage, and 4% undergoing necrosis. In comparison, the treated sample (RSV-LPHNC) showed 44% viable cells, 17.9% in early apoptosis, 34.5% in late apoptosis, and 3% necrotic cells.

 Our study, clearly demonstrated an increase in apoptotic progression from early to late apoptotic phases by RSV-LPHNC. One point specifically noted that RSV-LPHNC therapy selectively induced apoptosis rather than inducing non-specific necrotic cell death as seen by the modest and relatively equal necrosis levels (~3–4%) in both groups. Overall, the results show that RSV-LPHNC efficiently causes U87 glioblastoma cells to undergo apoptosis, especially promoting late-stage apoptotic cell death. Statistical analysis (unpaired Student’s t-test, n = 3) confirmed that RSV-LPHNC significantly increased late apoptosis compared to the control group (*P* < 0.05).

###  Reactive Oxygen Species Estimation

 Intracellular ROS levels in U87 glioblastoma cells were assessed by flow cytometry, and the results are summarized in Table 1 and Figure 2. The unstained group showed minimal background fluorescence (0.07% FITC-A⁺ cells; MFI 4.04), while control cells exhibited basal ROS levels (63.90% FITC-A⁺ viable cells; MFI 1228.17). Treatment with RSV-LPHNC caused a significant increase in ROS, with 70.51% FITC-A⁺ viable cells and an MFI of 1520.23, indicating enhanced oxidative stress. Elevated ROS is known to induce mitochondrial dysfunction, damage cellular macromolecules, and activate intrinsic apoptotic pathways.^27^ These results suggest that RSV-LPHNC promotes glioblastoma cell death through ROS-mediated oxidative stress, which aligns with the cytotoxicity and apoptosis findings observed in our study (Figure S1 (B)). Overall, RSV-LPHNC appears to exert its anticancer effects, at least in part, by increasing intracellular ROS and triggering apoptosis in U87 cells.

**Table 1:** ROS estimation statistics for unstained, control and RSV-LPHNC treated cells

**Sr. No**		**Events**	**% of Vis**	**Mean**
		**Unstained statistics**		
1	All events	9927	100.00	4.04
2	FITC-A+ Viable cells	7	0.07	295.84
		**Control statistics**		
3	All events	9939	100.00	827.22
4	FITC-A+ Viable cells	6351	63.90	1228.17
		**RSV-LPHNC statistics**		
5	All events	9941	100.00	1108.85
6	FITC-A+ Viable cells	7009	70.51	1520.23

**Figure 2:**
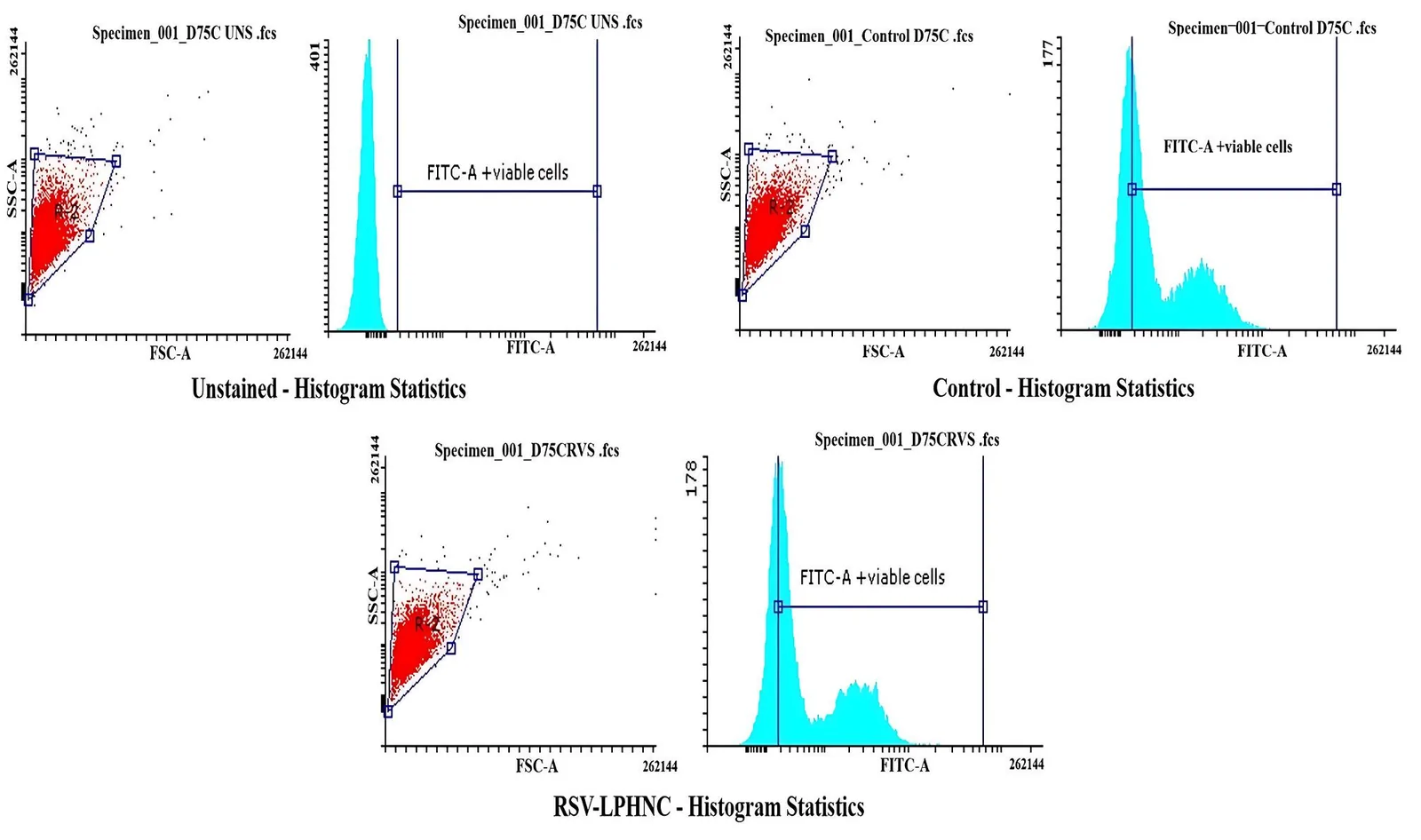
ROS estimation by flow cytometry

 The ROS estimation statistics in U87 cells reveal a significant elevation in reactive oxygen species (ROS) following RSV-LPHNC treatment compared to the control and unstained groups. One-way ANOVA followed by Tukey’s post hoc test indicated that the increase in ROS levels in RSV-LPHNC-treated cells was statistically significant compared to control (*P* < 0.01, n = 3).

 Based on the analysis of ROS Estimation of U87 cell line, it is observed that MFI Positive cells were present in Sample- RSV-LPHNC (15.29%) with respect to Control (12.355%) (Figure S1 (B)).

###  Elucidation of Kinetics of Cell Cycle Through Flow Cytometry

 The effect of RSV-LPHNC therapy on the distribution of cell cycle in U87 type of cells when compared to the control is demonstrated by the flow cytometry analysis shown in Figure 3. The G1 phase is represented by H2, the S phase by H3, and the G2/M phase by H4. These histogram data show the various stages in cell cycle. The control sample exhibited a balanced distribution of cells in G1-G2 phases, along with notable proportion in the S phase (H3), suggesting active DNA synthesis.

**Figure 3:**
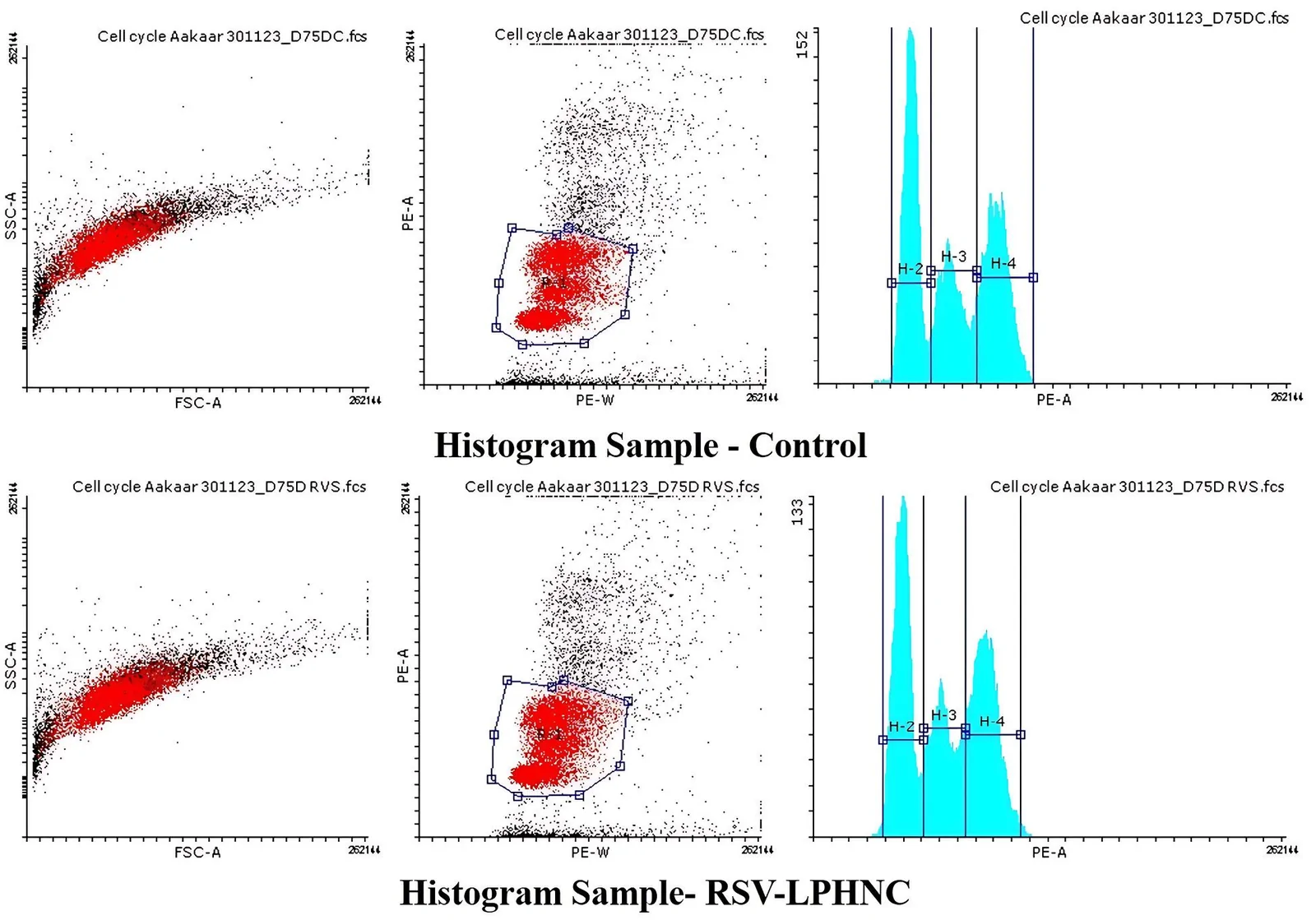
Elucidation of cell cycle kinetics through flow cytometry (Where H-2 = G1 Phase, H-3 = S Phase, H-4 = G2 Phase)

 There is an obvious change in the cell cycle profile after RSV-LPHNC therapy. A significant decrease in phase S population indicates that synthesis of DNA is being inhibited.^28^ At the same time, there seems to be a buildup of cells in the G1 phase (H2), which is a sign of cell cycle arrest before DNA replication. This G1 arrest might be caused by DNA damage or oxidative stress mediated by RSV-LPHNC, which triggers cell cycle checkpoints. Further evidence for the interruption of normal cell cycle progression comes from the reduced G2/M population (H4).^29^ All things considered, our findings imply that RSV-LPHNC causes a notable change in cell cycle dynamics, namely by stopping cells in the G1 phase and preventing them from entering the S phase. Its cytotoxic or anti-proliferative actions on glioblastoma U87 cells might be attributed to this mechanism.

 Cell cycle distribution of U87 glioblastoma cells was analyzed to evaluate the effect of RSV-LPHNC treatment (Figure S1 (C)). In control cells, 29.35% were in the G1 phase, 18.79% in the S phase, and 25.90% in the G2 phase. RSV-LPHNC-treated cells showed 29.48% in G1, 18.49% in S, and 27.75% in G2. Statistical analysis indicated that these differences were not significant (*P* > 0.05). Although the increase in G2 phase is modest, it may reflect slight accumulation of cells before mitosis, suggesting minor cell cycle modulation. Overall, these results indicate that RSV-LPHNC does not substantially alter cell cycle progression in U87 cells. Importantly, the cytotoxic effects of RSV-LPHNC appear to be primarily mediated via ROS-induced apoptosis, as supported by the ROS estimation and apoptosis assay results.

###  MMP Estimation with Flow Cytometry- U87

 The effect of RSV-LPHNC treatment on U87 MMP, as determined using flow cytometer, is shown in Figure 4. Red fluorescence (R) is more prevalent in the control group (upper panels), suggesting that live cells have intact mitochondrial membrane potential. On the other hand, there is a discernible change in the population in the RSV-LPHNC-treated group (bottom panels), with a higher percentage of cells located in the R3 area, which stands for depolarized mitochondria. Considerable decrease of MMP, a symptom of early apoptosis, is indicated by the treated cells’ increased green fluorescence (R3 area). Mitochondrial depolarization triggers downstream apoptotic signaling pathways and interferes with the synthesis of cellular energy.^30^ Thus, our finding implies that RSV-LPHNC causes mitochondrial malfunction in U87 cells, which aids in the intrinsic (mitochondrial-mediated) process of apoptosis induction. These results demonstrate that RSV-LPHNC can target the mitochondrial integrity of glioblastoma cells, potentially making it an effective therapeutic agent.

**Figure 4:**
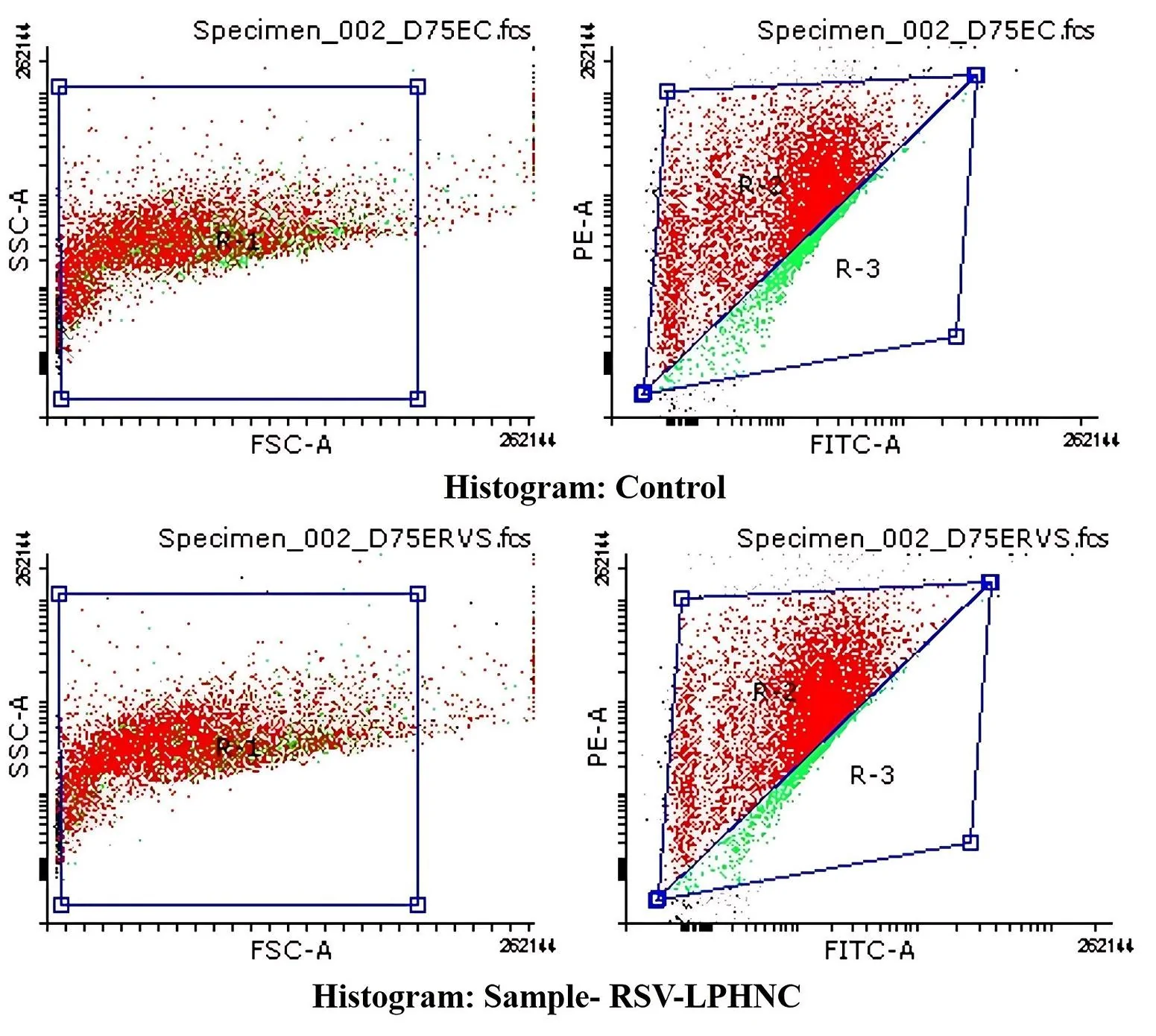
MMP Estimation with Flow Cytometry

 As per results it can be seen that RSV-LPHNC showed 105 % Relative MMP (low) with respect to control (100%) while 70.48 % Relative MMP (high) with respect to control (100%) (Figure S2 (A (i, ii))). Quantitative analysis (n = 3) revealed that the loss of MMP in RSV-LPHNC-treated cells was statistically significant compared to control (*P* < 0.05)

###  DNA Fragmentation Assay

 The findings of the DNA fragmentation test, which was performed on U87 cells to assess apoptosis brought on by different doses of the test drug, are shown in Figure 5. The molecular weight reference is the DNA ladder lane (L), which displays distinct bands. Normal cellular integrity and the lack of apoptosis are shown by the intact genomic DNA in the healthy control group (lane C), which shows no signs of fragmentation. Conversely, the treated samples A (619.3 µM), B (309.65 µM), and C (1238.6 µM) exhibit distinctive DNA laddering patterns that are suggestive of late-stage apoptosis’s hallmark of internucleosomal DNA breakage. Lane C (1238.6 µM) exhibits the maximum fragmentation, followed by A and B. The intensity and clarity of the fragmented bands increase with concentration. The RSV-LPHNC successfully triggers apoptosis in U87 cells via a concentration-dependent mechanism, as seen by this dose-dependent increase in DNA fragmentation. These findings confirm the compound’s potential as a treatment agent against glioblastoma by indicating that it activates apoptotic pathways to produce its cytotoxic effects. One-way ANOVA confirmed that DNA fragmentation increased significantly in a dose-dependent manner compared to control (*P* < 0.01, n = 3).

**Figure 5:**
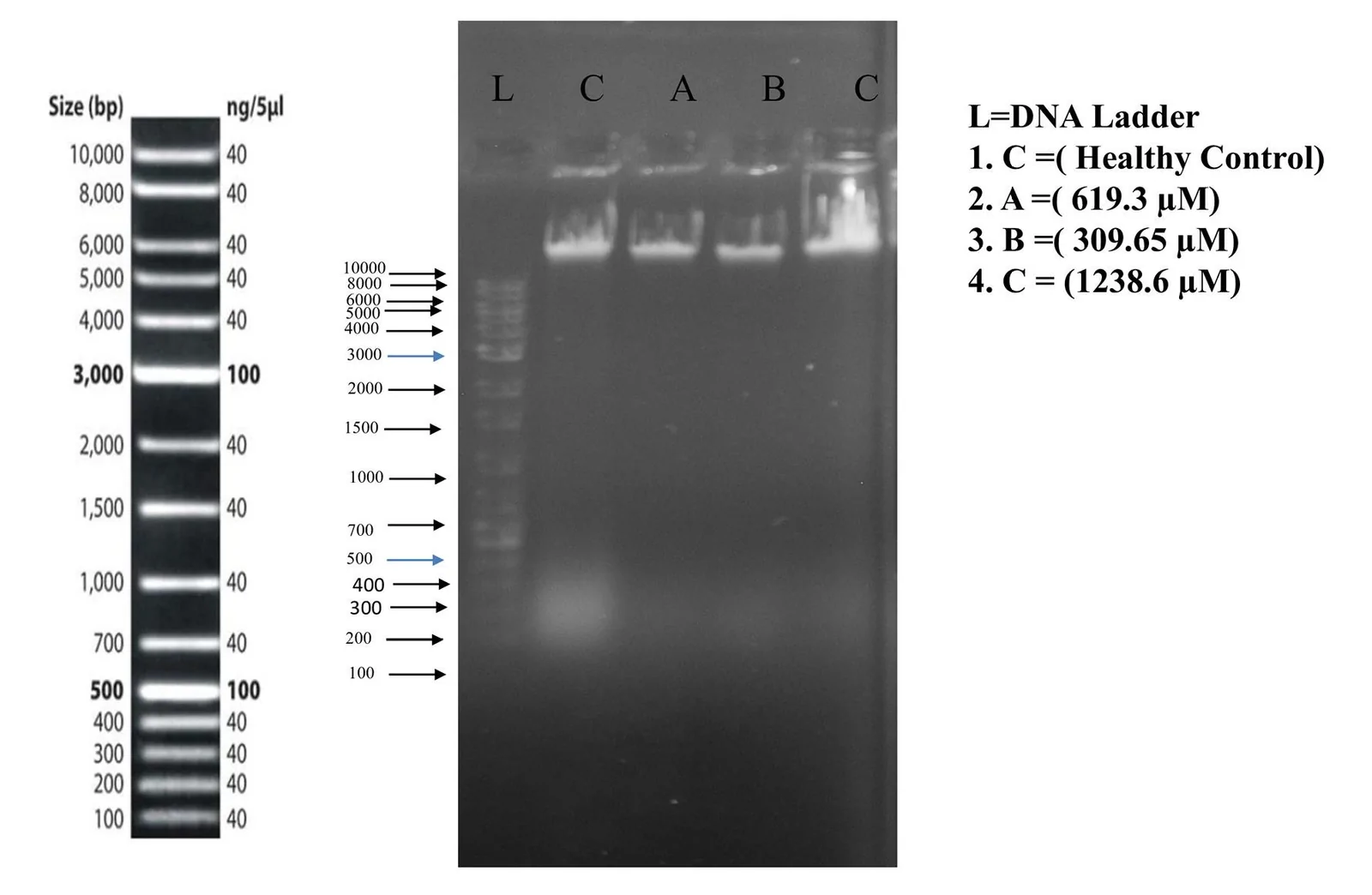
DNA Fragmentation Assay CL - U87

 It can be seen that U87 cells with several conc. of the sample in dose dependent manner, DNA fragmentation was observed to be elevated with rise in concentration of the sample. Specifically, the sample (RSV-LPHNC) showed lowest DNA fragmentation (58.98%) at 309.65 µM and highest DNA Fragmentation (134.42%) at 1238.6 µM with respect to control (cells without Treatment) (Figure S2 (B)).

###  In Vivo Pharmacokinetic Studies in Rat

 The comparative pharmacokinetic study demonstrated plasma concentration profile from suspension (RSV-SUSP) and (RSV-LPHNs) in rats. The significant enhancement in pharmacokinetic parameters were observed with RSV-LPHNs as compared to RSV suspension (Figure 6A). The marked rise in Cmax was observed from 3.45 µg/mL to 6.48 µg/mL while delay in Tmax from 2 to 4 hours suggested sustained release behavior (Table 2). Apart from this, the half-life (t_1/2_) of RSV was significantly increased from 1.87 to 8 hours with LPHNs, indicating extended circulation time.

**Figure 6:**
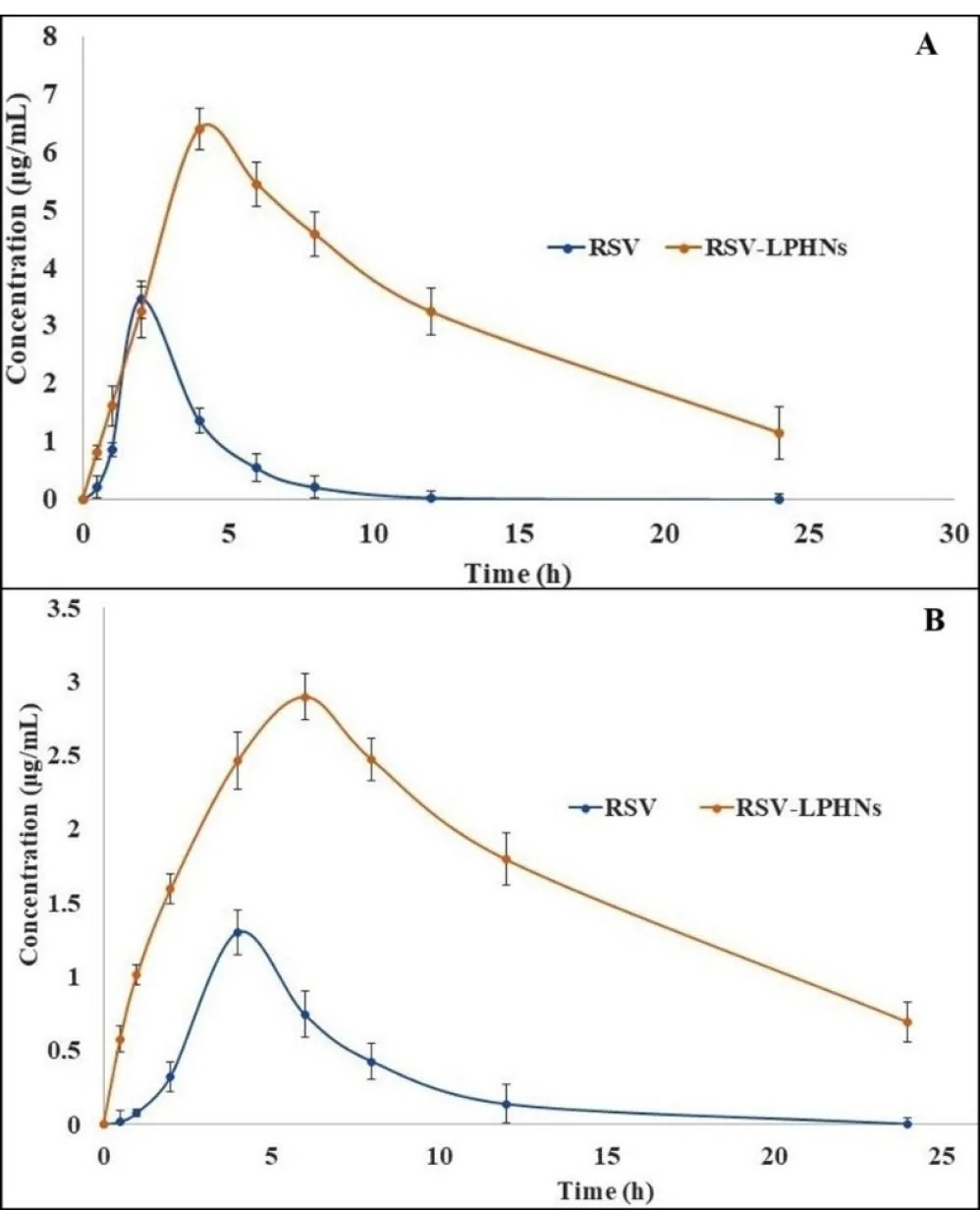
[A] Plasma concentration–time profiles of RSV in rats after oral administration of optimized RSV-LPHNs. Each data point represents the mean ± SD [B] In vivo study of RSV after per oral dose of RSV-SUSP and optimized RSV-LPHNs. Each data point represents the mean ± SD

**Table 2:** Pharmacokinetic parameters of RSV in rat plasma following oral administration

**Parameters **	**RSV**	**RSV-LPHNs**
t_1/2 _(h)	1.879 ± 0.19	8.901 ± 0.33
T_max_ (h)	2	4
C_max_ (μg/mL)	3.45 ± 0.71	6.48 ± 0.28
AUC_ (0-∞)_ (μg·h/mL)	10.682 ± 1.98	94.588 ± 2.12
MRT (0–1) (h)	3.48 ± 0.37	14.942 ± 0.67
CL (mL/h/kg)	1.872 ± 0.017	0.211 ± 0.008
Relative bioavailability	-	8.85

 The AUC (0–∞) for RSV-LPHNs (94.58 µg·h/mL) was substantially higher than that of the plain RSV (10.68 µg·h/mL), showing in a 3.77-times rise in relative bioavailability. Furthermore, the mean residence time (MRT) increased from 3.48 to 14.94 hours, and the clearance (CL) was significantly reduced from 1.87 to 0.21 units, confirming improved systemic retention and reduced elimination. These findings demonstrate that RSV-LPHNs enhance the oral bioavailability and sustain the release of resveratrol, making them a promising system to deliver drug with maximum therapeutic effectiveness (Figure 6A). Statistical analysis (unpaired Student’s t-test, n = 6) showed that the differences in Cmax, AUC, t1/2, and clearance between RSV suspension and RSV-LPHNC were statistically significant (*P* < 0.01)

###  Brain Distribution Study

 This was performed to interpret the quantity of RSV accumulated in the brain. The maximize brain concentration would be recommended for efficient treatment in Glioblastoma multiforme (GBM) which is very aggressive as well as malignant brain tumor.^31^ This study prominently showed enhanced delivery as well as sustained accumulation of RSV brain tissues of rat when administered as RSV-LPHNs as compared to the RSV suspension. Though, RSV-LPHNs showed higher Cmax (2.9 µg/mL) as compared to RSV suspension (1.3 µg/mL), RSV-LPHNs demonstrated significant prolonged half-life (t_1/2_) of 9.60 hours as compared to 2.49 hours, and delayed Tmax of 6 hours in comparison to 4 hours, indicating a sustained release profile (Table 3). The notable marked increase in AUC (0–∞) was observed from 7.11 to 47.20 µg·h/mL demonstrating 2.28-fold enhancement in relative bioavailability in brain tissue. Apart from these parameters, the MRT was enhanced from 6.38 to 17.27 hours while clearance (CL) was decreased from 2.81 to 0.42 units indicating prolonged retention and sustained elimination of RSV from brain tissues (Figure 6B).

**Table 3:** Pharmacokinetic and targeting parameters of RSV in rat brain tissues following oral administration.

**Parameters **	**RSV**	**RSV-LPHNs**
t_1/2 _(h)	2.49 ± 0.23	9.60 ± 0.17
T_max_ (h)	4	6
C_max_ (μg/mL)	1.3 ± 0.44	2.9 ± 0.75
AUC_ (0-∞)_ (μg·h/mL)	7.115 ± 1.33	47.207 ± 2.63
MRT (0–1) (h)	6.388 ± 0.27	17.278 ± 0.48
CL (mL/h/kg)	2.81 ± 0.043	0.423 ± 0.009
Relative bioavailability	-	6.634

 The findings of the present study confirmed that RSV-LPHNs has significantly improved the brain pharmacokinetic parameters as well as brain targeting of RSV, making them a promising strategy for neurotherapeutic applications. Unpaired Student’s t-test (n = 6) demonstrated that RSV-LPHNC significantly improved Cmax, AUC, and half-life in brain tissue compared to RSV suspension (*P* < 0.01).

## Conclusion

 The present study demonstrates that RSV-LPHNs effectively inhibit U87 glioblastoma cell growth by inducing apoptosis through ROS generation and mitochondrial dysfunction, alongside modulation of cell cycle progression. DNA fragmentation analysis confirmed a concentration-dependent apoptotic effect. Pharmacokinetic and brain distribution studies further revealed enhanced oral bioavailability, sustained release, and improved brain targeting of RSV via LPHNs. These findings highlight the novelty of RSV-LPHNs as a promising nanocarrier system with potential for targeted therapeutic application in glioblastoma, supporting its future translation in neuro-oncology.

## Competing Interests

 No conflict of interest.

## Data Availability Statement

 Data can be made available from corresponding author on reasonable request.

## Ethical Approval

 All animal experiments were conducted in strict accordance with the ethical norms as recognized by the Committee for the Purpose of Control and Supervision of Experiments on Animals (CPCSEA), Government of India and was approved by the Institutional Animal Ethics Committee (IAEC) of Trans-Genica Services Pvt. Ltd. (IEAC No:2105/PO/RcBt/S/20/CPCSEA).

## Supplementary File

Supplementary file contains Figures S1 and S2.
