# Supplementary material for: Enhancing Apoptosis in Glioblastoma Through Hybrid Nanoparticle-Mediated Resveratrol Delivery: In Vitro Cytotoxicity and in Vivo Pharmacokinetic Study
Source: Adv Pharm Bull. 2026 May 9;16(3):573–82. doi: 10.34172/apb.46117 (PMC13745442; doi:10.34172/apb.46117)
Supplement: Supplementary file 1 [file apb-16-573-s001.pdf]

Adv Pharm Bull 2026;16(3):573-S1
doi:10.34172/apb.46117
https://apb.tbzmed.ac.ir

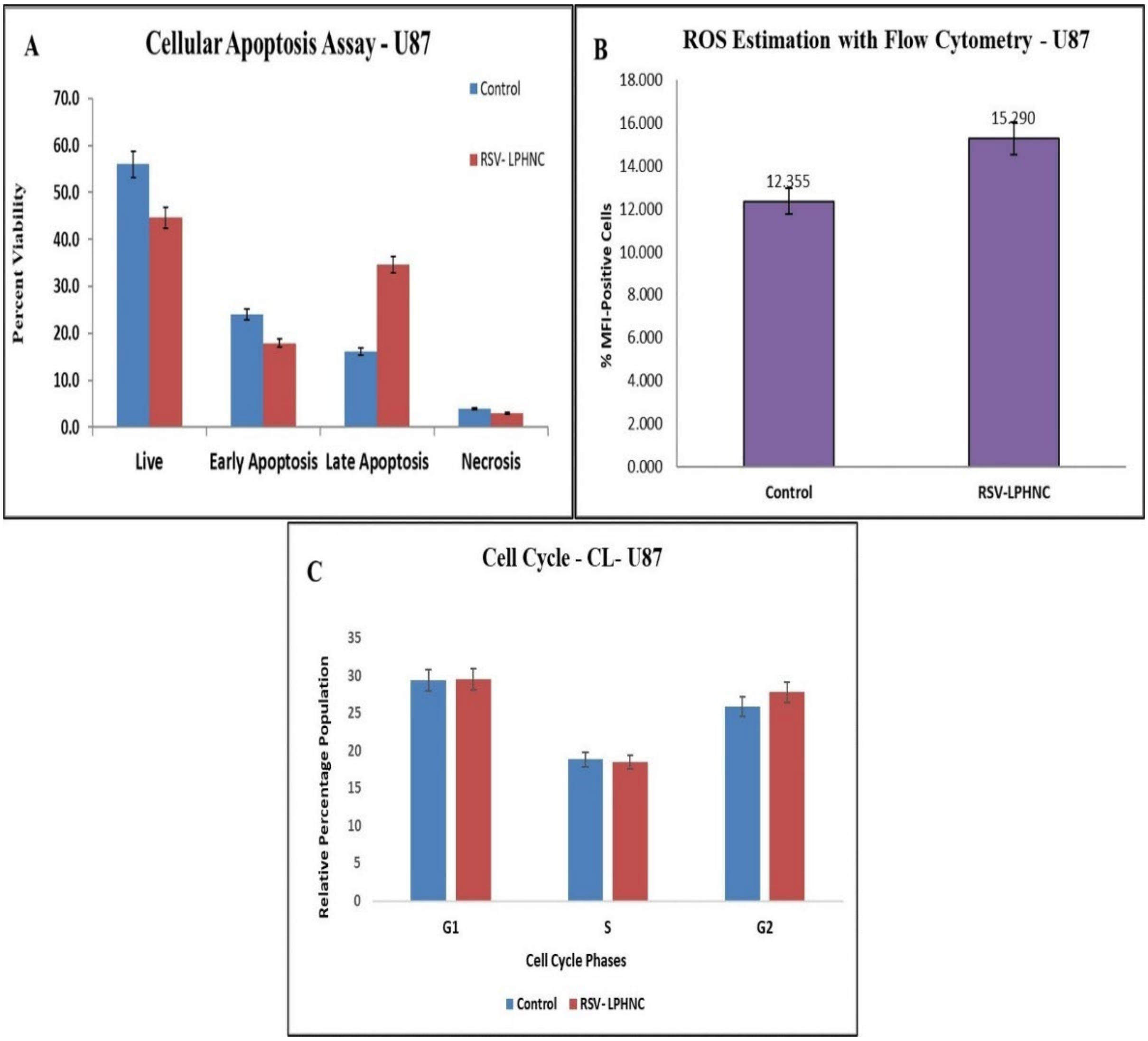

Figure S1. Comparative cellular apoptosis assay between control and RSV-LPHN. [B] ROS Estimation of U87 cell line with Flow cytometry. [C] Elucidating cell cycle kinetics considering cell cycle phases.

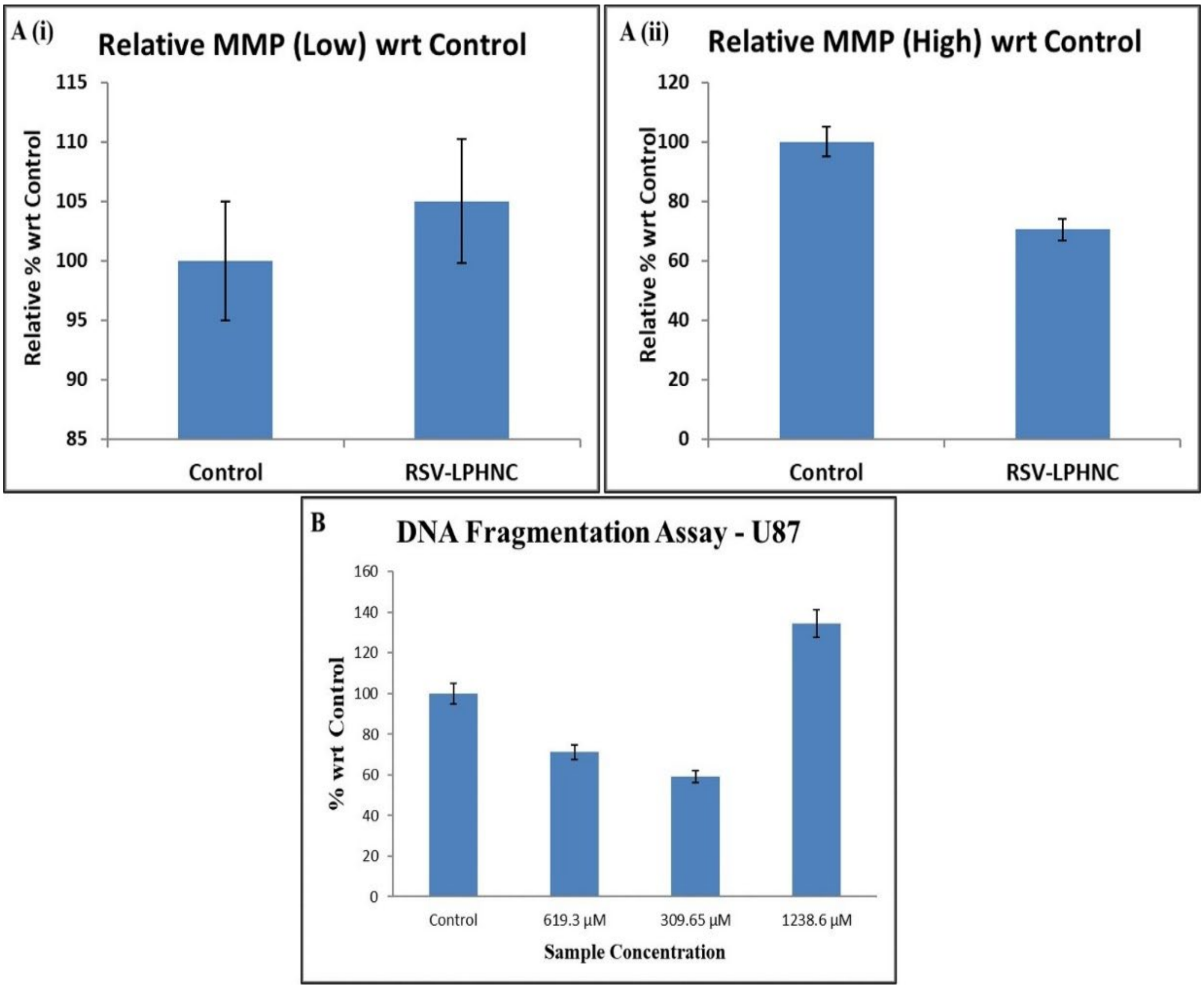

Figure S2. [A (i, ii)] Relative MMP (Low and High) with respect to control. [B] DNA fragmentation assay with respect to concentrations.
